# Society Meetings

**Published:** 1895-12

**Authors:** 


### Society Meetings.

Buffalo Academy of Medicine.—The section on gynecology and obstetrics holds its meetings the fourth Tuesday of every month. The remainder of the program until the vacation season is as follows:

December 24, 1895, discussion: (*a*) Surgical treatment of puerperal sepsis, Dr. C. C. Frederick; (*b*) What are the contra-indications to operation? Dr. P. W. Van Peyma; (*c*) What are the results thus far of operative treatment, and what are the causes of failure in fatal cases? Dr. H. E. Hayd.

January 28, 1896: Points of interest culled from my gynecological practice, Dr. Marcel Hartwig. Paper: subject to be announced, Dr. S. Y. Howell.

February 25th: A few points of interest in gynecology in relation to insanity, Dr. Helene Kuhlman. Paper: subject to be announced, Dr. H. E. Hayd.

March 24th: How classify retroflexion with a view to treatment? Dr. Edmund Luke. Placenta previa, Dr. T. G. Allen. Paper: subject to be announced, Dr. C. C. Frederick.

April 28th: Paper: subject to be announced, Dr. M. D. Mann. Septicemia in the new-born, Dr. Irving M. Snow.

May 26th: Digestive disturbances in gynecological and obstetrical cases, Dr. A. L. Benedict. Some cases in practice, Dr. John J. Walsh.

June 23d: Phlegmasia Alba Dolens, Dr. Ludwig Schroetter. Paper: subject to be announced, Dr. M. A. Crockett.

Meetings are called to order at 8.30 P. M. Fellows of the Academy are requested to present specimens and report cases. Papers to occupy thirty minutes and the general discussions ten minutes.

DR. CARLTON C. FREDERICK, *Pres.*
DR. LILLIAN C. RANDALL, *Sec'y.*

---

THE Southern Surgical and Gynecological Association held a most interesting meeting at Washington, November 12, 13 and 14, 1895. The president, Dr. Lewis McLane Tiffany, of Baltimore, administered the affairs of the association with dignity and discretion.

Dr. S. C. Busey, of Washington, in delivering an address of welcome, said :

I solicit your aid and coöperation in our effort to secure the protection of our people from the horde of imposters and charlatans, which you have driven from your borders by the enactment and enforcement of medical practice laws, and which has made the District of Columbia a common rendezvous where the most atrocious methods of the charlatan and mercenary impositions are openly and flagrantly committed to the wrong, injury and robbery of its citizens. You represent the most influential and intelligent class of suffragists for whose aid on the hustings and at the polls we plead. To state the deplorable condition of this district fully and broadly, there are five medical schools and several medical societies chartered by acts of Congress, or under the general incorporation law authorised and empowered to license persons to practise the art and science of medicine, without any uniform, and by some, without any standard of qualification, beyond the ability and willingness of the applicant to pay the required fees or give promissory notes for such payment ; and under the provisions of the general incorporation law, any dozen of persons can obtain a charter upon payment of the fee for recording the same, authorising them as a body corporate to confer the degree of M. D. at their pleasure and will. Such is the status of this Federal territory, which is under the exclusive jurisdiction of the highest tribunal of legislation in the land, made up of the representatives and senators from forty-nine states and territories, which have enacted medical practice laws for the protection and welfare of their citizens. Take these facts home with you and re-echo them throughout the length and breadth of the land, that such criminal neglect, not less disgraceful and scandalous than the slums of vice, may not continue to afflict the citizens of the Federal territory.

A reception was held by the medical profession of Washington for the association at the Arlington on Tuesday evening, and Dr. Joseph Taber Johnson entertained the association at his residence on Wednesday evening. The next meeting was appointed to be held at Nashville the second Tuesday in November, 1896, and Dr. Ernest S. Lewis, of New Orleans, was elected president.

---

THE Tri-State Medical Society, (Iowa, Illinois and Missouri,) at its last meeting elected the following officers: president, Dr. Robert H. Babcock, Chicago; first vice-president, Dr. A. H. Cordier, Kansas City; second vice-president, Dr. W. A. Todd, Chariton, Ia.; treasurer, Dr. C. S. Chase, Waterloo, Ia.; secretary, Dr. G. W. Cale, St. Louis. The next meeting will be held in Chicago the first Tuesday, Wednesday and Thursday in April, 1896.

